# Supplementary material for: Neuropsychological Characterization of Aggressive Behavior in Children and Adolescents with CD/ODD and Effects of Single Doses of Medications: The Protocol of the Matrics_WP6-1 Study
Source: Brain Sci. 2021 Dec 11;11(12):1639. doi: 10.3390/brainsci11121639 (PMC8699463; doi:10.3390/brainsci11121639)
Supplement: Supplementary file 1 [file brainsci-11-01639-s001.zip › brainsci-1430255-supplementary.pdf]

Supplementary materials

Table S1. Matrics WP6-1 study schedule of events

	Screening	Baseline [a] (case & control)		Single-blind, placebo controlled, acute dose, cross-over, randomised medication challenge [b]		
**Visit**	**-1**	**0a**	**0b**	**1**	**2**	**3**
**Week**	**-4 to -1**	**0**		**1**	**2**	**3**
Informed Consent / Assent	✓	✓				
Inclusion / Exclusion Criteria	✓	✓				
Socio-demographics	✓					
Family medical history	✓					
Medical and psychiatric history	✓	✓				
Medication History	✓					
Current medications & Confirm washout/prohibited medicines	✓	✓		✓	✓	✓
Physical Examination	✓			✓	✓	✓
Height	✓	✓				
Weight	✓	✓		✓	✓	✓
Body Temperature	✓	✓		✓	✓	✓
Vital Signs (BP, HR)	✓	✓		✓ [c]	✓ [c]	✓ [c]
Pregnancy test	✓	✓		✓	✓	✓
K-SADS	✓					
WISC-IV/WAIS-IV	✓					
TRF	✓					
YSR	✓					
CBCL	✓					
NCBRF-TIQ	✓					
MOAS	✓					
CPRS	✓					
BRIEF	✓					
ICU	✓					
CGI-S	✓					
C-GAS	✓					

Visit	-1	0a	0b	1	2	3
**Week**	**-4 to -1**	**0**		**1**	**2**	**3**
Risk assessment for pharmacological treatment	✓	✓		✓		
Randomization (Group A/B)		✓				
Randomization (Drug/Placebo sequence)				✓		
Drug/placebo administration				✓	✓	✓
E4 wristband (HR and skin conduct)		✓	✓	✓	✓	✓
Salivar Cortisol level		✓	✓	✓	✓	✓
IED [d]		✓				
FEERT [e]		✓				
DD [e]		✓				
MJ [e]		✓				
PD [e]		✓				
RVIP [d]			✓	✓	✓	✓
DMS [d]			✓	✓	✓	✓
PRT [e]			✓	✓	✓	✓
NCGT [e]			✓	✓	✓	✓
FA go no go [e]			✓	✓	✓	✓
RLT [e]			✓	✓	✓	✓
ToM [e]			✓	✓	✓	✓
UG [e]			✓	✓	✓	✓
Adverse events reporting				✓	✓	✓

a Screening procedures have been performed on two subsequent different days within Week 0
b Only CD/ODD
c Before drug administration and after the end of the testing session
d CANTAB battery
e EMOTICOM battery
